# Supplementary material for: A gene-expression module identifies circulating immune cells with enhanced recruitment to sites of inflammation
Source: iScience. 2025 Nov 26;29(1):114227. doi: 10.1016/j.isci.2025.114227 (PMC12794507; doi:10.1016/j.isci.2025.114227)

iScience, Volume *29*

Supplemental information

A gene-expression module identifies circulating immune cells with enhanced recruitment to sites of inflammation

Debajyoti Sinha, Thomas Laurent, Alexis Broquet, Cynthia Fourgeux, Thibault Letellier, Gaelle Tilly, Sarah Bruneau, Simon Ville, Laurence Bouchet-Delbos, Julien Brancherau, Clarisse Kerleau, Sophie Brouard, Gilles Blancho, Magali Giral, Regis Josien, Richard Danger, Antoine Roquilly, Nicolas Degauque, and Jeremie Poschmann

Supplemental Legends & Figures

Figure S1 ScRNA-seq integration of a cohort of kidney allograft recipients

A) Expression profiles of cell-specific markers distinguish the PBMC populations. Average expression is the log-normalized expression average of the cells by cell type, size of the dots is associated to the fraction of cells of the cluster in which the gene is detected.

B) Stacked barchart showing cell type composition (% of each celltype is indicated)

C) Cell type proportion by cluster in the overall PBMC population. Comparison include Stable patients, Rejection (ABMR+TCMR) and two public datasets: healthy volunteers from Stephenson et al, 2021, and healthy volunteers from Reyes et al, 2020. One-way ANOVA with Tukey's multiple comparisons post hoc test was performed, *P < 0.05, **P < 0.01, ***P < 0.001, ****P < 0.0001.

D & E) UMAP projection showing the sample distribution, ABMR=Humoral rejection, TCMR=Cellular rejection, STA=Stable, TOL=Tolerant. Second UMAP shows the timepoint distribution, T0=Time point 0 (Graft), T1=Time point 1, T2=Time point 2.

F) Stacked Barcharts of cell type composition stratified by condition (left) or batch (right)

G) K-bet acceptance rate by cell types following the CCA batch integration. Complete k-bet acceptance rate was computed on the overall PBMC population.

Figure S2 Assessing module distribution

A) Alluvial plot showing the shared origin of each module across batches. Each cell is assigned to the most enriched module they express.

B) Boxplots showing the distribution of Pearson correlation coefficient *R* of pairwise gene expression correlation by cell type across modules. A module of random genes was generated to compare with the nine other modules. Multiple gene pairs from each module are selected to compute the rho values using a subset of common cells within the cell type. 1000 repetitions of 50 pairs each yielded 50000 *R* values, as represented in every box. The subset of common cells changes with every repetition.

C) Boxplots showing the distribution of Pearson correlation coefficient *R* of pairwise gene expression correlation by cell type for Module 9.

D) Super violin plots showing the longitudinal trend of module 6 in NK, CD4, CD8 T cells, CD14 and B cells, stratified by individuals from each batch.

Figure S3 Cellular characterization of pig PBMC by scRNA-seq analysis

A) Image J analysis of immuno-stained slices to quantify cell infiltration. Red boxes show counting areas which excludes glomeruli. Right panel shows the cells which were then counted using Image J software.

B) Pig data were annotated using the Sscrofa reference genome and associated to their human homologue. The expression profiles of cell type-specific markers is shown in the dotplot of relative expression by cell type. Size of the dots are associated to the fraction of cells of the cluster in which the gene is detected. The UMAP embeds the 4 samples (D0, D2, D4, D6) with their corresponding cell type annotation.

Fig S4 Transwell assay experiments

A) Quantification of CXCR4 protein expression on T cells before and after migration, shown as box-and-whisker plots summarizing four independent replicates.

B) Gating strategy used in the transwell assay for cell type annotation. Lymphocytes were gated based on forward scatter (FSC) and side scatter (SSC), followed by singlet selection and the identification of CD3+ T cells. CD4+ and CD8+ T cells were further delineated based on CD45RA and CCR7 expression to distinguish between naïve (Tn), central memory (Tcm), and effector memory RA (TemRA) subsets. Additionally, CD4 T regulatory (Treg) cells were identified by CD25 and CD127 expression to differentiate between activated (aTreg) and non-Treg populations.

B) Percentage of CD4+ and CD8+ T cells expressing CD69 under five different experimental conditions: before the transwell assay (baseline), CXCL12 only, HDMEC alone, CXCL12 with HDMEC, and after migration through HDMEC with CXCL12.
C) UMAP visualization of scRNA-seq data from the transwell experiment showing all cell populations. The top plot displays T cell subtypes identified by their transcriptional profiles, including CD4+ and CD8+ T naïve cells, CD8+ T effector memory RA (TEMRA) cells, mucosal-associated invariant T (MAIT) cells, and CD4+ and CD8+ T effector memory (TEM) cells annotated using Celltypist. The bottom plot shows the distribution of cells based on their experimental condition: control which is CXCL12-, CXCL12+ and transmigrated. The clustering of cells in the UMAP space indicates differences in transcriptional states based on both cell type and condition.
D) ALARM gene expression by condition. Dot plot showing the relative expression levels of ALARM-associated genes in CD4+ and CD8+ T cells across the different conditions of the transwell assay (Migrated, CXCL12+, and CXCL12-). The size of the dots represents the percentage of cells expressing the gene, and the color intensity represents the level of expression, with darker shades indicating higher expression.

Fig 5S. Overview of the cohort distribution
A) ALARM module score distribution by cell type based on scRNA-seq analysis of PBMC from 45 unrelated healthy individuals[40].
B) Cell type proportions of each individual summarized by boxplots
C) Left: ALARM module score distribution across sex. No significance was found for the sex parameter with a t-test. Right: ALARM module score distribution across age categories. No significance was found for the age parameter with a one-way ANOVA test.
D) Permutation-based resampling analysis of ALARM module expression in PBMCs following LPS injection. For each comparison and cell type, 200 cells were randomly sampled from each group 5,000 times, and Wilcoxon rank-sum tests were performed in each iteration. Histograms show the frequency distribution of –log10(p-values) across permutations. Comparisons include Healthy vs. LPS_90mins (orange) and LPS_90mins vs. LPS_10hours (blue). The proportion of permutations with FDR-adjusted p-values <0.05 is indicated for each condition.
E) As in D, permutation-based resampling analysis of ALARM module expression in PBMCs from patients with bacteremia-associated sepsis (Bac–SEP) or leukocyte-infiltrating urinary tract infection (Leuk–UTI). Comparisons are shown for each cell type (Bac–SEP vs. control in blue, Leuk–UTI vs. control in orange). The percentage of permutations yielding FDR-adjusted p-values <0.05 and the mean Wilcoxon p-value across permutations are reported.

Fig 6S. ALARM genes can classify immune-mediated diseases
A) Distribution of the bulk RNA-seq samples across the 28 circulating immune cell types for the 337 patients distributed across 10 immune mediated diseases and the 79 healthy controls.
B) Area Under Curve (AUC) and Cohen's Kappa score across cell types in disease and healthy patients.
C) AUC and Cohen's Kappa score across cell subtypes in disease and healthy patients
D) Distribution of the ratio of genes associated to Gene-Disease Association (GDA) terms in 1000 modules of 61 randomly selected genes. The ALARM module has a ratio of 0.39 genes associated to GDA terms (red line).

Supp. Figure 1

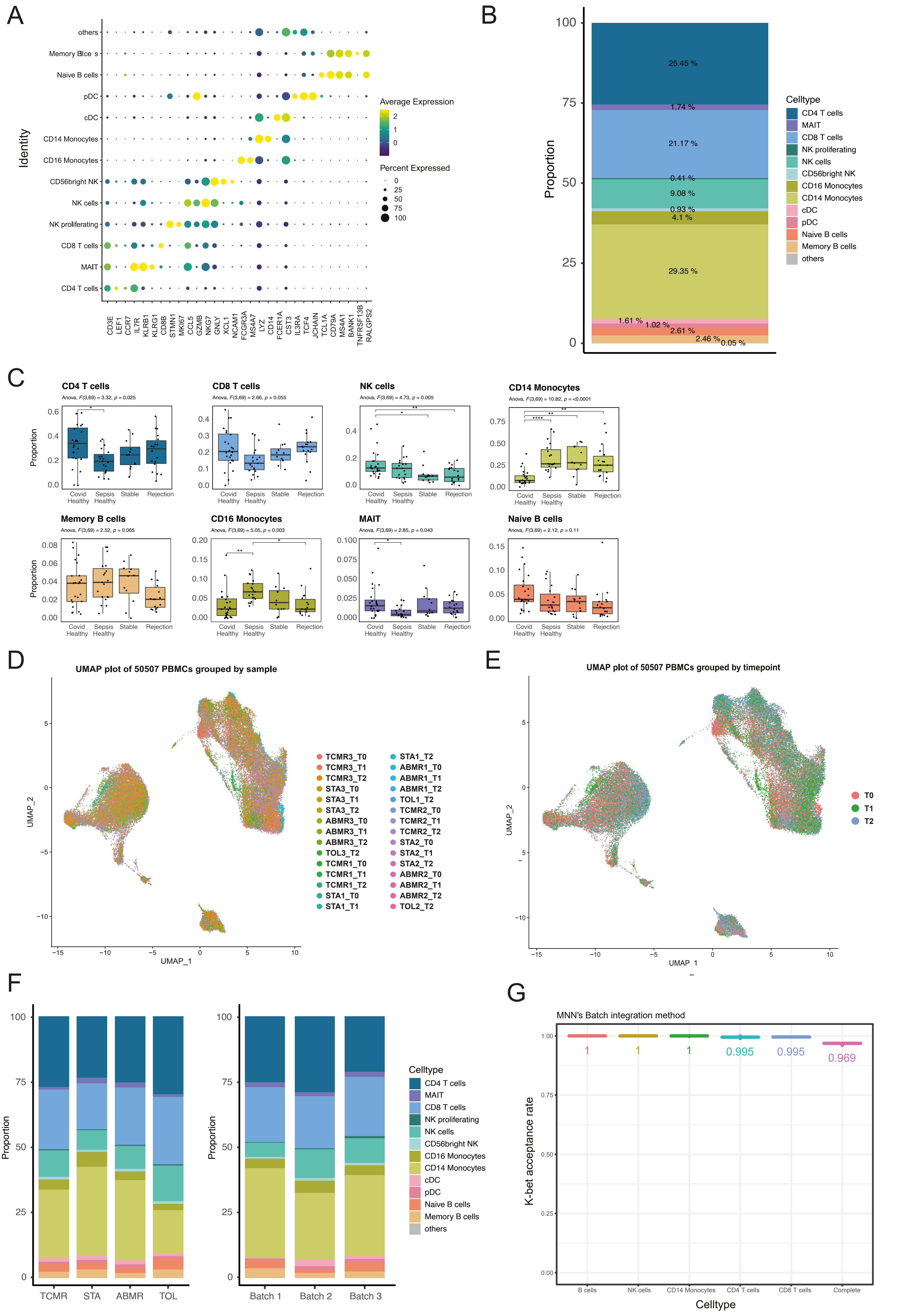

Supp. Figure 2

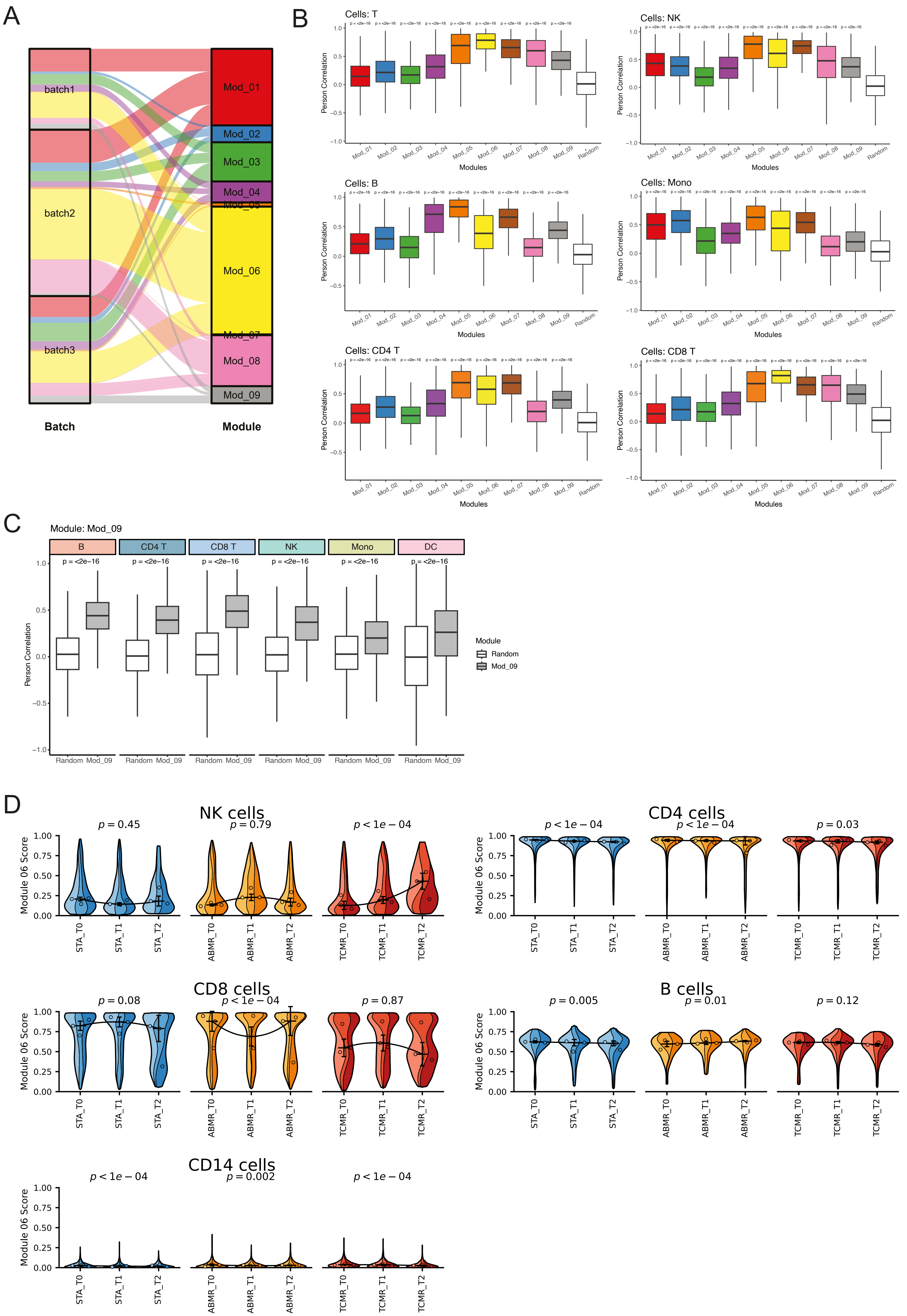

Supp. Figure 3

A

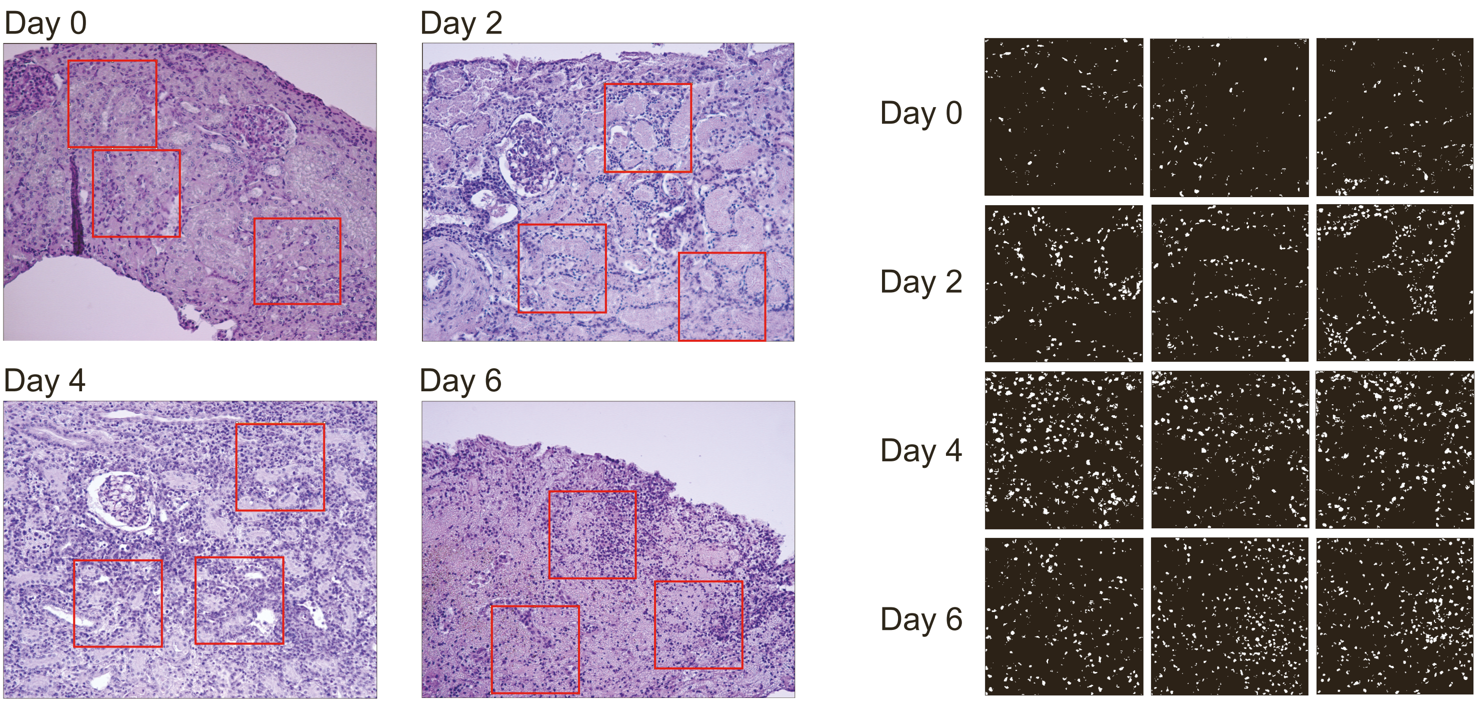

B

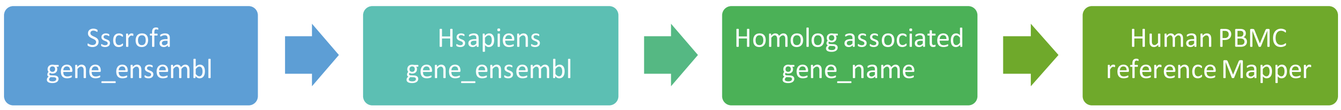

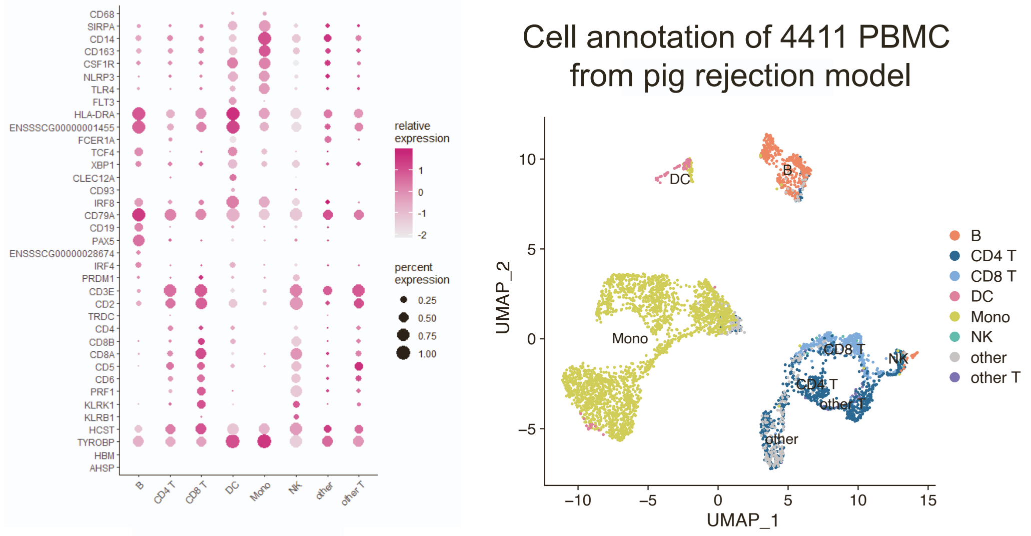

Supp. Figure 4

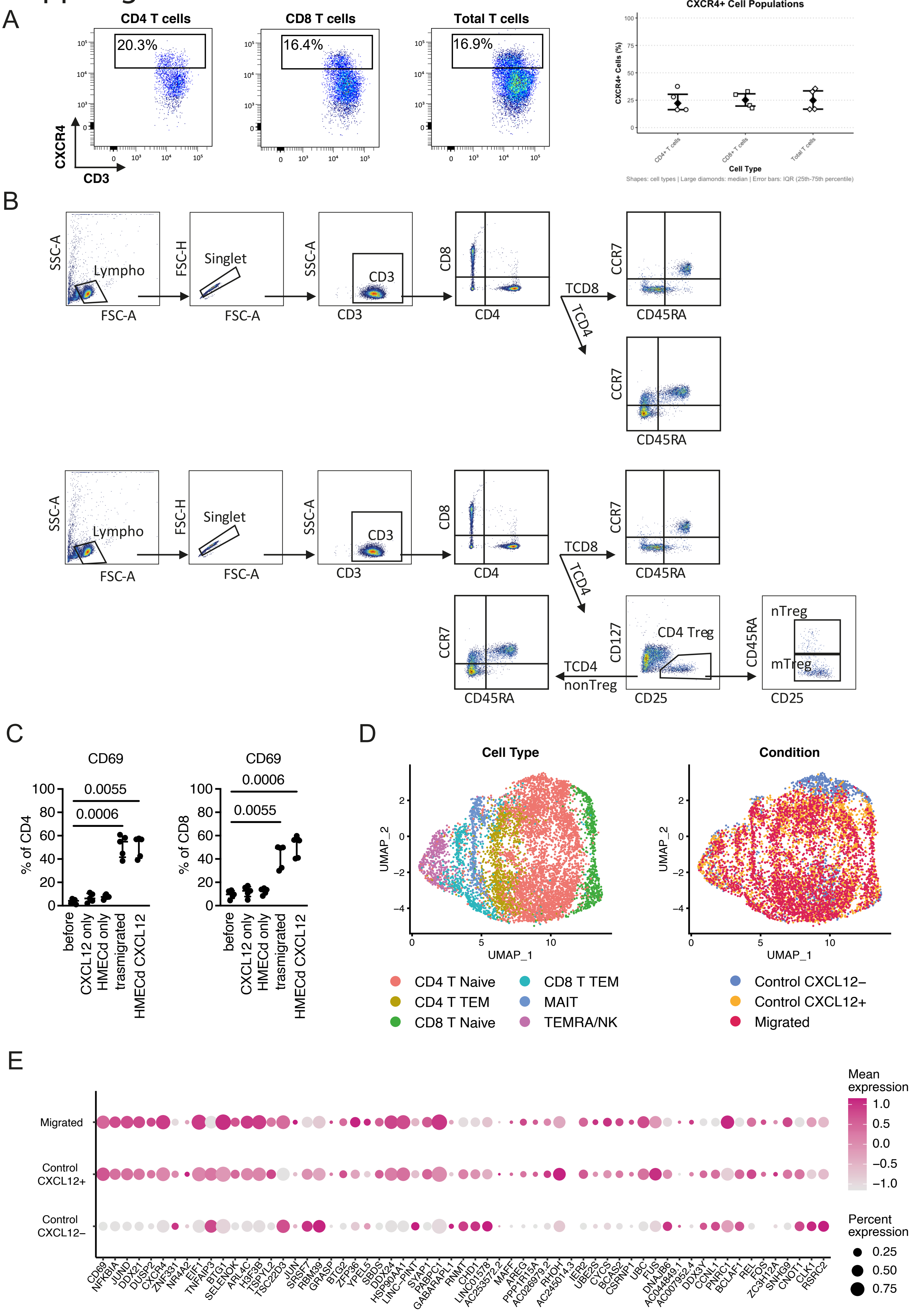

Supp. Figure 5

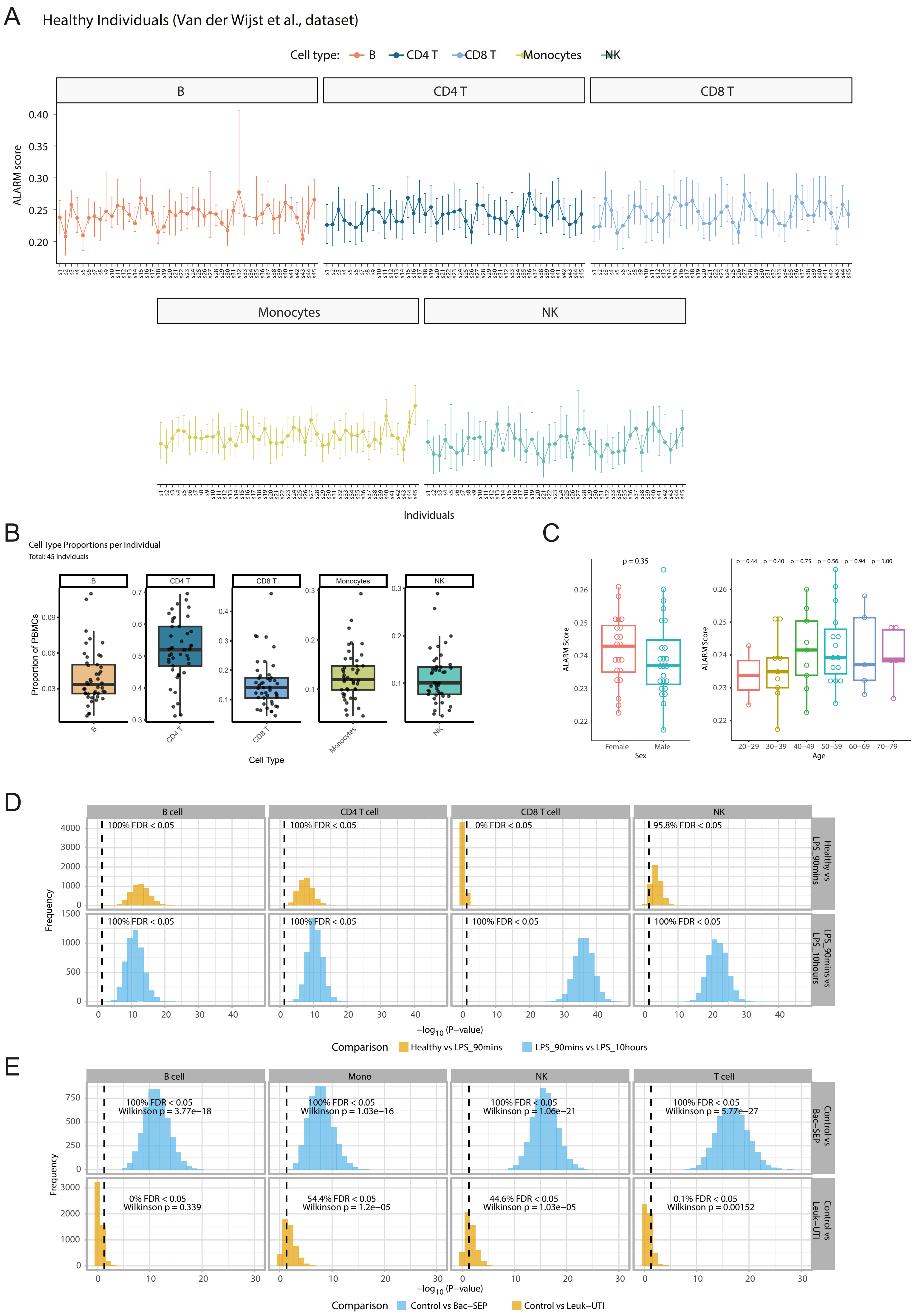

Supp. Figure 6

A

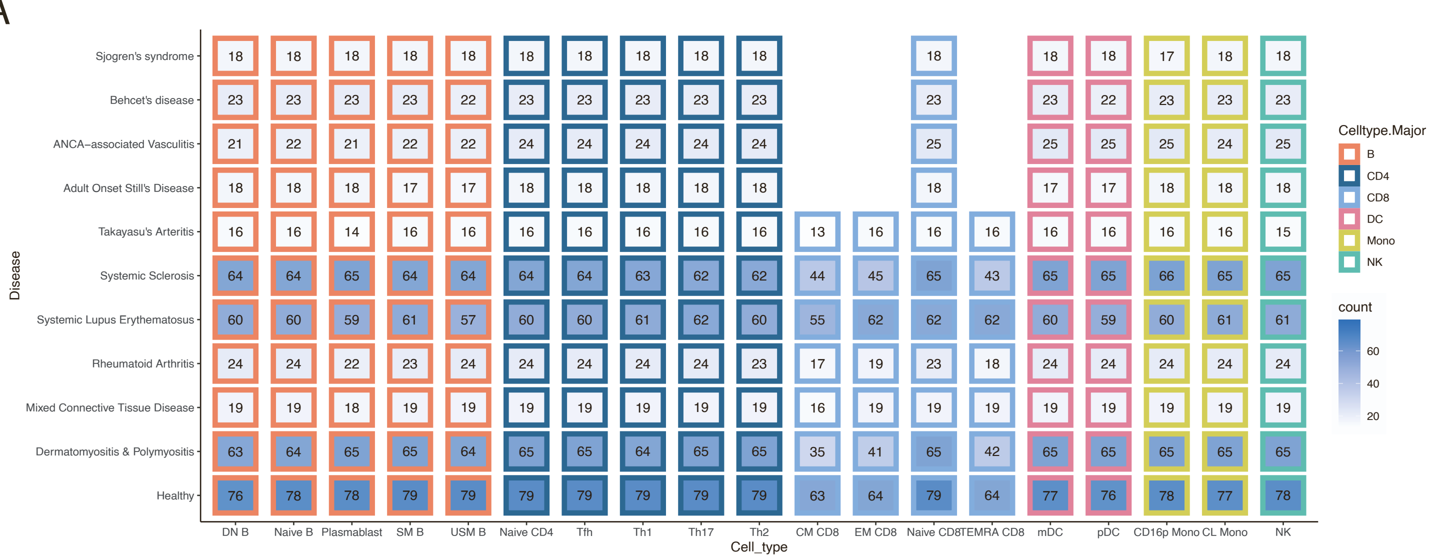

B

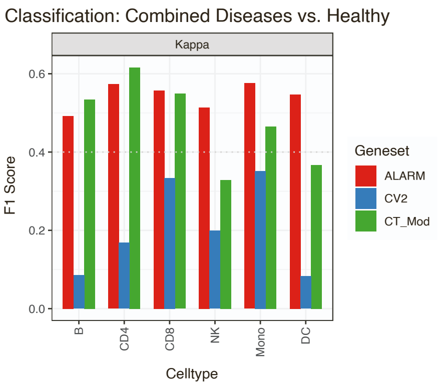

C

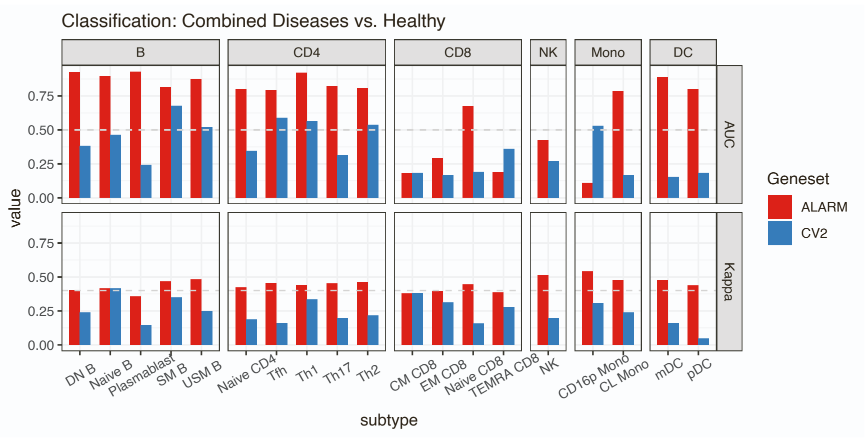

D

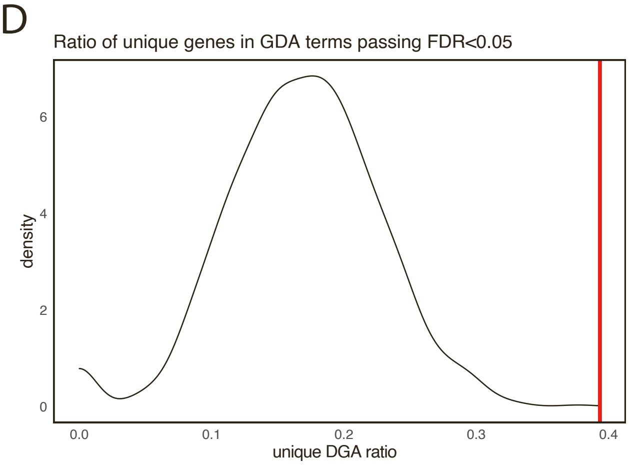

Supplement: Document S1. Figures S1–S6 [file mmc1.pdf]
